# Public Health Response to an Avian Influenza A(H7N8) Virus Outbreak in Commercial Turkey Flocks — Indiana, 2016

**DOI:** 10.15585/mmwr.mm6748a2

**Published:** 2018-12-07

**Authors:** Jennifer A. Brown, Reema Patel, Lynn Maitlen, Donna Oeding, Karen Gordon, Joshua L. Clayton, Shawn Richards, Pam Pontones, James Brewer, Sara Blosser, Joan Duwve

**Affiliations:** ^1^Indiana State Department of Health; ^2^Dubois County Health Department, Jasper, Indiana.

## Abstract

In January 2016, highly pathogenic avian influenza (HPAI) A(H7N8) virus and low pathogenicity avian influenza (LPAI) A(H7N8) virus were detected in commercial turkey flocks in Dubois County, Indiana. The Indiana State Department of Health (ISDH) and the Dubois County Health Department (DCHD) coordinated the public health response to this outbreak, which was the first detection of HPAI A(H7N8) in any species (*1*). This response was the first to fully implement unpublished public health monitoring procedures for HPAI responders that were developed by the U.S. Department of Agriculture (USDA) and CDC in 2015 (Sonja Olsen, CDC, personal communication, October 2017). No cases of zoonotic avian influenza infection in humans were detected during the outbreak.

## Investigation and Results

On January 15, 2016, ISDH was notified by the Indiana State Board of Animal Health that HPAI A(H7N8) virus had been confirmed in a commercial turkey flock in Dubois County, Indiana. In accordance with USDA guidelines (*2*), the State Board of Animal Health promptly began active surveillance for HPAI in commercial poultry flocks within a radius of 6.2 miles (10 km) of the infected premises. By January 16, avian influenza H7 virus was detected in nine additional commercial turkey flocks; eight of these were confirmed as LPAI A(H7N8), and testing was inconclusive for one. Two additional poultry flocks were classified as dangerous contact premises because of their proximity to infected premises (*2*). The circulating HPAI and LPAI strains were suspected to be closely related; the State Board of Animal Health therefore elected to depopulate all 10 avian influenza H7-infected premises and both dangerous contact premises (a total of 414,223 birds). Depopulation was accomplished primarily by premises owners and industry representatives with the assistance of volunteer offenders from the Indiana Department of Correction (IDOC), whose participation was approved by the IDOC deputy commissioner. Offenders were compensated using the standard IDOC pay schedule and underwent medical clearance, N95 respirator fit testing, and job-specific training that included information about the zoonotic potential of avian influenza viruses. Depopulation was completed by January 16 for the index flock and by January 20 for the remaining flocks. The majority of poultry carcasses were disposed of by in-barn composting, and infected premises were cleaned and disinfected (*2*,*3*). Repopulation of all infected premises and dangerous contact premises was permitted as of May 1.

ISDH and DCHD recommended that responders be monitored during the response and for 10 days after their last possible exposures for influenza-like illness (ILI), defined as either 1) self-reported fever with cough or sore throat, or 2) conjunctivitis with or without additional symptoms. All responders received instructions to seek medical attention and contact public health authorities if they developed ILI during the 10-day period. USDA monitored federal employees, contractors, and subcontractors who participated in the response. ISDH and DCHD monitored state and local responders, using adapted, unpublished USDA/CDC public health monitoring procedures for HPAI A(H5) that were first circulated in September 2015 and later updated in November 2015. Responders were classified into three risk categories: 1) no risk, 2) low but not zero risk, and 3) some risk. Responders with low but not zero risk were those exposed to infected or potentially infected birds or their environments while using appropriate personal protective equipment (PPE) (*3*) as well as persons who were not exposed to birds or their environments but who worked or resided on infected premises; these persons were contacted by telephone on their first and last day of monitoring. Responders with some risk were those exposed to infected or potentially infected birds or their environments who did not use appropriate PPE or had a breach in PPE; these persons were actively monitored, with daily contact by visit, telephone call, text, or e-mail.

Although it is difficult to estimate the total number of responders, the number of daily on-site personnel peaked at 516 on day 4 of the response, most of whom were federal employees or contractors. DCHD, IDOC, and other local health departments conducted risk assessment and monitoring for 166 state and local responders. These included 93 farm workers or residents, 67 officers and offenders from state correctional facilities, and six local USDA employees who had completed their response activities. Among these 166 responders, 74 (45%) were monitored for some risk exposures, 67 (40%) were monitored for low but not zero risk exposures, four (2%) were monitored with no risk status recorded, seven (4%) had no exposure, five (3%) declined to be monitored, and nine (5%) were lost to follow-up. Among the 145 persons who were monitored, 14 (10%) reported current or recent ILI symptoms, 12 (86%) of whom were tested during January 16–22, 2016, with a median of 1 day from onset to medical evaluation. Nine patients had nasopharyngeal/oropharyngeal specimens collected, one had a conjunctival specimen collected, and two had both types of specimens collected. Specimens were tested for influenza A virus by real-time reverse transcription–polymerase chain reaction at the ISDH Laboratories; all 12 patients tested negative for influenza A virus.

## Public Health Response

On January 15, 2016, Indiana activated its Emergency Operations Center with staffing consistent with the Federal Emergency Management Agency Emergency Support Functions (ESFs) appropriate to the response (ESF-1 = Transportation, ESF-5 = Emergency Management, ESF-8 = Public Health and Medical, ESF-10 = Oil and Hazardous Waste, ESF-11 = Food, Agriculture and Natural Resources, and ESF-13 = Law Enforcement).* The Indiana State Board of Animal Health was the lead state agency for this response, and ISDH provided public health and medical services support. DCHD led the local response, including monitoring for exposed county residents, in cooperation with Memorial Hospital in Jasper, Indiana. The State Incident Management Team was deployed to Dubois County to establish a unified command post in conjunction with USDA. ISDH deployed a field liaison to the unified command post to communicate with other state and local agencies. ISDH supported several missions from the state Emergency Operations Center, including distribution of N95 respirators and laboratory testing supplies and placement of the antiviral medication oseltamivir at the local hospital.

ISDH’s major actions during the public health response included developing a demobilization packet for responders, issuing a Health Alert Network advisory to Dubois County and surrounding counties with recommendations for health care providers, establishing syndromic surveillance queries in the Indiana Public Health Emergency Surveillance System to detect community-acquired cases, and developing recommendations for the use of antivirals.

## Discussion

Public health monitoring procedures for H7N8 responders were successfully implemented during this outbreak; no cases of zoonotic avian influenza infection were detected. The risk for zoonotic transmission in this outbreak was thought to be low at the time. No human infections with influenza A(H7N8) viruses had ever been reported,^†^ and preliminary genetic analyses did not suggest enhanced virulence or transmission in mammals (*4*). However, other avian influenza H7 viruses have caused human infections, including severe respiratory illnesses (*5*,*6*), and human coinfection with an avian influenza A virus and a human influenza A virus presents a theoretical risk for emergence of a novel influenza A virus through genetic reassortment (*7*). The HPAI virus in this outbreak was suspected to have emerged as a result of spontaneous mutation in a circulating LPAI virus; this hypothesis was supported by later genetic analyses (*1*). A study conducted after the outbreak found that the HPAI virus exhibited enhanced virulence in mouse and ferret models, but that only the LPAI strain was transmissible; however, transmissibility in mammals and capacity to rapidly acquire increased virulence is a concerning combination of characteristics (*8*).

The unpublished USDA/CDC public health monitoring procedures that were adapted for use in this outbreak were developed for responders to an HPAI A(H5) outbreak. HPAI and LPAI are differentiated based on genetic features and the extent to which these viruses produce morbidity and mortality in poultry (*9*). The classification does not predict the probability of zoonotic transmission or severity of human illness (*6*); in fact, LPAI is capable of causing severe morbidity and mortality in humans (*10*). Given this and that many responders worked on both HPAI-infected and LPAI-infected premises, the same guidance was used for all H7N8-infected flocks.

The USDA/CDC public health monitoring plan is currently being updated (James Kile, CDC, personal communication, August 2018). The updated plan will allow for passive monitoring of persons wearing PPE and responding to certain influenza A H5 and H7 viruses that have caused outbreaks in the United States but have no history of causing human infections (e.g., the H7N8 virus described in this report). The updated plan will also cover all avian influenza viruses of public health concern, including both HPAI and LPAI viruses.

Several challenges to human health monitoring were identified during this outbreak. First, receipt of contact information for responders by the local health department was delayed in the initial stages because of the urgency and complexity of the animal health response. Complete information for exposed responders was not received by DCHD until 5 days into the response (on January 20), although preliminary information was provided earlier. Second, tears in Tyvek suits were reportedly common because of the nature of animal handling activities; this could have resulted in misclassification of disease exposure risk. Finally, mobilization of a large number of responders within a short period raised concerns that PPE and monitoring recommendations were not being implemented consistently.

Enhanced communication and information sharing among local, state, and federal agencies would improve identification of exposed persons and coordination of specimen collection, testing, and medical care for ill responders. This could be accomplished by 1) effectively communicating public health needs and recommendations to all stakeholders in the response; 2) identifying processes for early identification of exposed persons (e.g., badging systems); 3) designating a local/state/federal public health department liaison to be embedded in the unified command post to facilitate coordinated implementation of human health monitoring, including obtaining names and contact information for responders; and 4) conducting daily debriefings with safety officers in the incident command system to identify injuries or breaches in PPE that could elevate responder risk. In future outbreaks, ISDH will also recommend that responders to outbreaks of AI viruses of public health concern entering the exclusion zone (hot zone) and contamination reduction zone (warm zone) in infected premises (*2*) undergo active monitoring for 10 days after the last date of exposure, whether or not they were wearing appropriate PPE. This adjustment is expected to facilitate identification of personnel requiring monitoring and ensure that even responders with unrecognized or unreported breaches in PPE will be monitored.

**Summary**

**What is already known about this topic?**

Prolonged or close contact with birds infected with avian influenza (AI) virus increases the risk for zoonotic infection in humans. Monitoring exposed persons for 10 days might facilitate early detection and reporting of zoonotic AI.

**What is added by this report?**

Monitoring procedures for highly pathogenic AI (HPAI) responders were successfully implemented during a 2016 outbreak of HPAI A(H7N8) in commercial turkey flocks in Indiana. No human cases of AI were identified.

**What are the implications for public health practice?**

Collaboration among local, state, and federal partners is essential during AI outbreak responses. Monitoring should be considered for all responders who had contact with infected birds or their environments, regardless of whether personal protective equipment was worn.

## Footnotes

* https://emilms.fema.gov/is230c/fem0104160text.htm.

† https://www.cdc.gov/flu/news/avian-influenza-h7n8-update.htm.

## References

[1] Killian ML, Kim-Torchetti M, Hines N, Yingst S, DeLiberto T, Lee DH. Outbreak of H7N8 low pathogenic avian influenza in commercial turkeys with spontaneous mutation to highly pathogenic avian influenza. Genome Announc 2016;4:e00457-16. 10.1128/genomeA.00457-16 27313288 PMC4911467

[2] US Department of Agriculture. Highly pathogenic avian influenza response plan: the red book. Riverdale, MD: US Department of Agriculture, Animal and Plant Health Inspection Service, Veterinary Services; 2017. https://www.aphis.usda.gov/animal_health/emergency_management/downloads/hpai_response_plan.pdf

[3] US Department of Agriculture. Highly pathogenic avian influenza. Riverdale, MD: US Department of Agriculture, Animal and Plant Health Inspection Service; 2018. https://www.aphis.usda.gov/aphis/ourfocus/animalhealth/emergency-management/fadprep-hpai

[4] US Department of Agriculture. Epidemiologic and other analyses of Indiana HPAI/LPAI-affected poultry flocks: March 18, 2016 report. Fort Collins, CO: US Department of Agriculture, Animal and Plant Health Inspection Service, Veterinary Services; 2016. https://www.aphis.usda.gov/animal_health/animal_dis_spec/poultry/downloads/Epidemiologic-Analysis-March-18-2016.pdf

[5] Belser JA, Bridges CB, Katz JM, Tumpey TM. Past, present, and possible future human infection with influenza virus A subtype H7. Emerg Infect Dis 2009;15:859–65. 10.3201/eid1506.090072 19523282 PMC2727350

[6] Kile JC, Ren R, Liu L, Update: increase in human infections with novel Asian lineage avian influenza A(H7N9) viruses during the fifth epidemic—China, October 1, 2016–August 7, 2017. MMWR Morb Mortal Wkly Rep 2017;66:928–32. 10.15585/mmwr.mm6635a2 28880856 PMC5689040

[7] Zhu Y, Qi X, Cui L, Zhou M, Wang H. Human co-infection with novel avian influenza A H7N9 and influenza A H3N2 viruses in Jiangsu province, China. Lancet 2013;381:2134. 10.1016/S0140-6736(13)61135-6 23769236

[8] Sun X, Belser JA, Pulit-Penaloza JA, Pathogenesis and transmission assessments of two H7N8 influenza A viruses recently isolated from turkey farms in Indiana using mouse and ferret models. J Virol 2016;90:10936–44. 10.1128/JVI.01646-16 27681133 PMC5110157

[9] Swayne DE, Pantin-Jackwood M. Pathogenicity of avian influenza viruses in poultry. Dev Biol (Basel) 2006;124:61–7. 16447495

[10] Kang M, Lau EHY, Guan W, Epidemiology of human infections with highly pathogenic avian influenza A(H7N9) virus in Guangdong, 2016 to 2017. Euro Surveill 2017;22:30568. 10.2807/1560-7917.ES.2017.22.27.30568 28703705 PMC5508330

